# AKAP6 inhibition impairs myoblast differentiation and muscle regeneration: Positive loop between AKAP6 and myogenin

**DOI:** 10.1038/srep16523

**Published:** 2015-11-13

**Authors:** Sae-Won Lee, Joo-Yun Won, Jimin Yang, Jaewon Lee, Su-Yeon Kim, Eun Ju Lee, Hyo-Soo Kim

**Affiliations:** 1 Biomedical Research Institute and IRICT, Seoul National University Hospital, 101 DaeHak-ro, JongRo-gu Seoul, 110-744, Republic of Korea; 2 Department of Internal Medicine and IRICT, Seoul National University Hospital, 101 DaeHak-ro, JongRo-gu Seoul, 110-744, Republic of Korea; 3 Department of Molecular Medicine and Biopharmaceutical Sciences, Graduate School of Convergence Science and Technology, Seoul National University, Korea

## Abstract

Skeletal muscle regeneration occurs continuously to repair muscle damage incurred during normal activity and in chronic disease or injury. Herein, we report that A-kinase anchoring protein 6 (AKAP6) is important for skeletal myoblast differentiation and muscle regeneration. Compared with unstimulated skeletal myoblasts that underwent proliferation, differentiated cells show significant stimulation of AKAP6 expression. AKAP6 knockdown with siRNA effectively halts the formation of myotubes and decreases the expression of the differentiation markers myogenin and myosin heavy chain. When shAKAP6-lentivirus is delivered to mice with cardiotoxin (CTX)-induced muscle injury, muscle regeneration is impaired compared with that of mice injected with control shMock-lentivirus. The motor functions of mice infected with shAKAP6-lentivirus (CTX+shAK6) are significantly worse than those of mice infected with shMock-lentivirus (CTX+shMock). Mechanistic analysis showed that AKAP6 promotes myogenin expression through myocyte enhancer factor 2A (MEF2A). Notably, myogenin increases AKAP6 expression as well. The results of chromatin immunoprecipitation and luciferase assays showed that myogenin binds to an E-box site on the AKAP6 promoter. Taken together, our findings demonstrate a novel interplay between AKAP6 and myogenin, and we suggest that AKAP6 is an important regulator of myoblast differentiation, myotube formation, and muscle regeneration.

Muscle damage occurs during normal activity and in response to chronic disease or injury. Skeletal muscle regeneration occurs continuously to repair this damage^1^,^2^ and is characterized by the proliferation and differentiation of muscle precursor cells followed by their fusion with one another or existing myofibers to form multinucleated myotubes^2^.

The process of skeletal muscle development is tightly regulated by the myogenic regulatory factor (MRF) family, which includes MyoD, Myf5, myogenin, and MRF4^1^. During embryonic development, specification of mesodermal precursor cells to the myogenic lineage requires the up-regulation of MyoD and Myf5, which are expressed in proliferative myogenic cells called myoblasts. Proliferating myoblasts terminally differentiate to myocytes that express the late MRFs (myogenin and MRF4). Mononuclear myocytes fuse with one another to form multinuclear myotubes/myofibers and express myofibrillar proteins such as myosin heavy chain (MyHC), the major structural protein in myotubes.

Scaffold proteins play a central role in the physical assembly of signaling components. Most scaffolds use a tethering mechanism to increase the efficiency of the interaction between partner molecules. These proteins also have a role in the allosteric modulation of the catalytic activity of kinases and phosphatases. Scaffold proteins regulate the efficiency and selectivity of pathways and orchestrate new responses from preexisting signaling components. Therefore, scaffold proteins are flexible platforms assembled via the mixing and matching of interaction molecules^3^. One family of well-studied scaffolding proteins is composed of the A-kinase anchoring proteins (AKAPs)^4^,^5^. AKAPs recruit protein kinase A (PKA) close to its substrate/effector proteins, directing and amplifying the biological effects of cAMP signaling. Although AKAPs were identified based on their binding to PKA, they also bind to other signaling molecules, mainly phosphatases and kinases, which regulate AKAP targeting and activate other signaling pathways^4^,^5^,^6^,^7^,^8^.

One of AKAPs, AKAP6 (also known as muscle AKAP or AKAP100), is highly expressed in the heart, skeletal muscle, and brain. It is localized to the perinuclear membrane in differentiating myoblasts and involved in anchoring PKA and cardiac ryanodine receptor to the nuclear membrane^5^,^9^,^10^. AKAP6 also binds to diverse signaling proteins such as the ryanodine receptor, PKA, Posphodiesterase4D3 (PDE4D3), ERK5, PP1, and PP2A. AKAP6 reportedly increases contractility and induces cardiomyocyte hypertrophy^5^,^11^. Therefore, AKAP6 not only localizes signaling enzymes to specific subcellular locations but also contributes to signal integration and cross-talk.

In this study, we found that AKAP6 is required for skeletal muscle regeneration. AKAP6 expression gradually increases along with the differentiation of both mouse C2C12 myoblasts and human skeletal muscle myoblasts (HSMMs). Furthermore, AKAP6 knockdown with siRNA blocks myogenic differentiation. Muscle regeneration is impaired when AKAP6 is blocked with a shAKAP6-lentivirus *in vivo*. Mechanistic analysis showed that AKAP6 and myogenin exist in a positive feedback loop. AKAP6 increases myogenin and promotes myotube formation, and myogenin increases AKAP6 expression.

## Results

### AKAP6 expression increased during skeletal myoblast differentiation

We investigated which AKAP proteins have roles in skeletal myoblast differentiation by examining the expression of the AKAP family proteins known to be related to muscles: AKAP6^11^, AKAP12^12^, AKAP-Lbc^13^, and AKAP79^14^.

Mouse C2C12 myoblasts were cultured in growth medium and then placed in differentiation medium. We first observed the morphological change of C2C12 myoblasts (Fig. S1). The formation of nascent myotubes appeared within 3 days, and more than 80% of the cells fused into matured myotubes on day 4. However, myotubes were rarely detected under proliferation conditions until day 4 (Fig. S1a). In parallel, protein extracts were analyzed with western blotting (Fig. S1b,c). Surprisingly, only AKAP6 increased upon differentiation. No differences in AKAP12, AKAP-Lbc, or AKAP79 were observed between proliferation and differentiation conditions, suggesting that AKAP6 is involved in skeletal myoblast differentiation.

**Figure 1:**
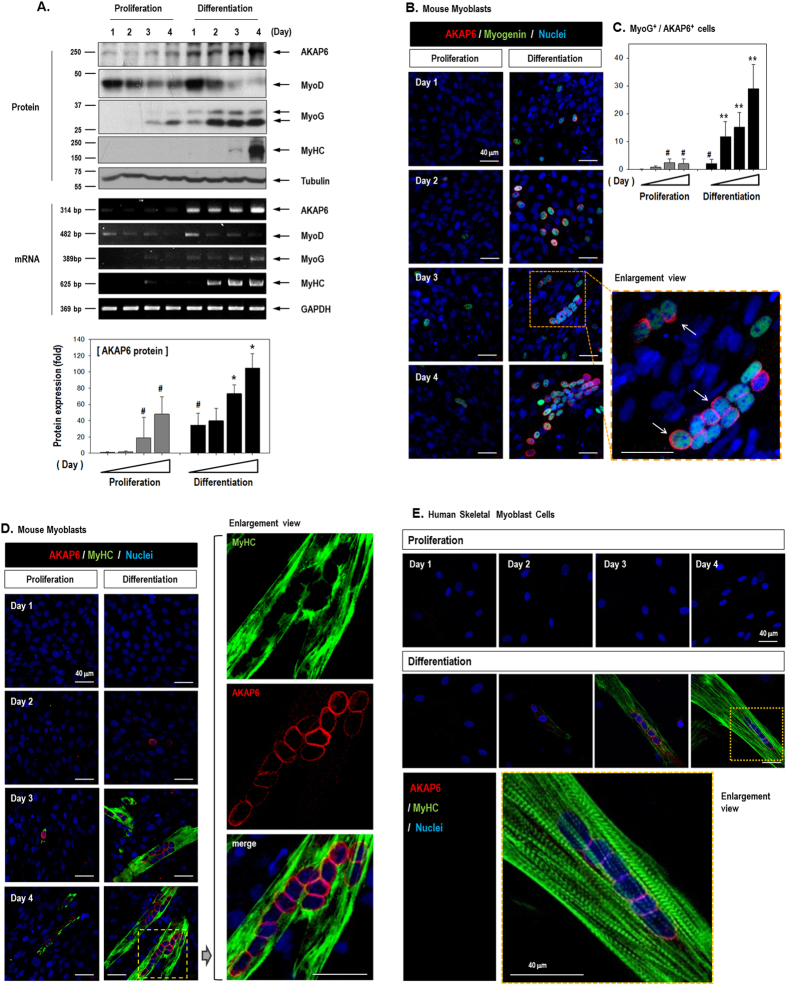
Expression pattern of AKAP6 and several differentiation markers in differentiating myoblasts. (**A**) Daily harvested protein and RNA samples from mouse C2C12 myoblasts analyzed by western blotting (Top) and RT-PCR (Middle). AKAP6 and the differentiation markers myogenin and MyHC increased during differentiation. The quantification graph shows the relative fold change compared with levels on day 1 under proliferative conditions (Bottom, n = 3). ^#^p < 0.05 versus proliferation on day 1; *p < 0.05 versus differentiation on day 1 (one-way ANOVA). (**B**) C2C12 cells were stained with AKAP6 (Red) and myogenin (Green). AKAP6 staining is observed in the nuclear envelope (arrows), and myogenin is detected in the nucleus (Magnification: ×400). (**C**) Quantification graph for AKAP6^+^/Myogenin^+^ cells (n = 5) under proliferation (gray) or differentiation (black) conditions. ^#^p < 0.05 versus proliferation on day 1; **p < 0.01 versus differentiation on day 1 (one-way ANOVA). (**D**) AKAP6-positive (Red) and MyHC-positive (Green) cells are mature myotubes (Magnification: ×400). (**E**) Immunofluorescence staining for AKAP6 (Red) and MyHC (Green) in human skeletal muscle myoblasts (HSMMs; magnification: ×400).

We further confirmed the expression pattern of AKAP6 during the differentiation of skeletal myoblasts by using several differentiation markers (Fig. 1). The differentiation of skeletal myoblasts to functional myotubes involves sequentially regulated transcription factors^1^,^15^. MyoD, the myogenic determination gene, is expressed in proliferating myogenic progenitors before the onset of myoblast differentiation^1^,^16^. Myogenin is then expressed upon myotube fusion, and the terminal differentiation gene, MyHC, is expressed several days after fusion^1^,^17^,^18^. We observed that MyoD was already expressed under the proliferation conditions, and MyoD was activated in response to differentiation signals and then progressively down-regulated (Fig. 1A). AKAP6 increased upon differentiation, and myogenin was coincidently up-regulated upon differentiation. MyHC, the terminal differentiation marker, was strongly up-regulated on differentiation day 4 (Fig. 1A, Fig. S2).

We performed immunofluorescence staining for AKAP6, myogenin, and MyHC to confirm the expression of AKAP6 both in response to a differentiation stimulus and in myotubes (Fig. 1B–D, Fig. S3). AKAP6 immunofluorescence was clearly observed in the nuclear envelope (red). Additionally, AKAP6 and differentiation markers myogenin (green) or MyHC (green) were co-expressed in the same subset of cells. The majority of these cells showed co-expression, and few cells expressed only one gene (Fig. S3). In addition, AKAP6 expression induced by the differentiation stimulus was confirmed in HSMMs (Fig. 1E). As in mouse cells, multinucleated differentiating cells showed AKAP6 staining. These data suggest that AKAP6 has a role in skeletal myoblast differentiation in both mice and humans.

### AKAP6 knockdown inhibits myoblast differentiation and myotube formation

**Figure 2:**
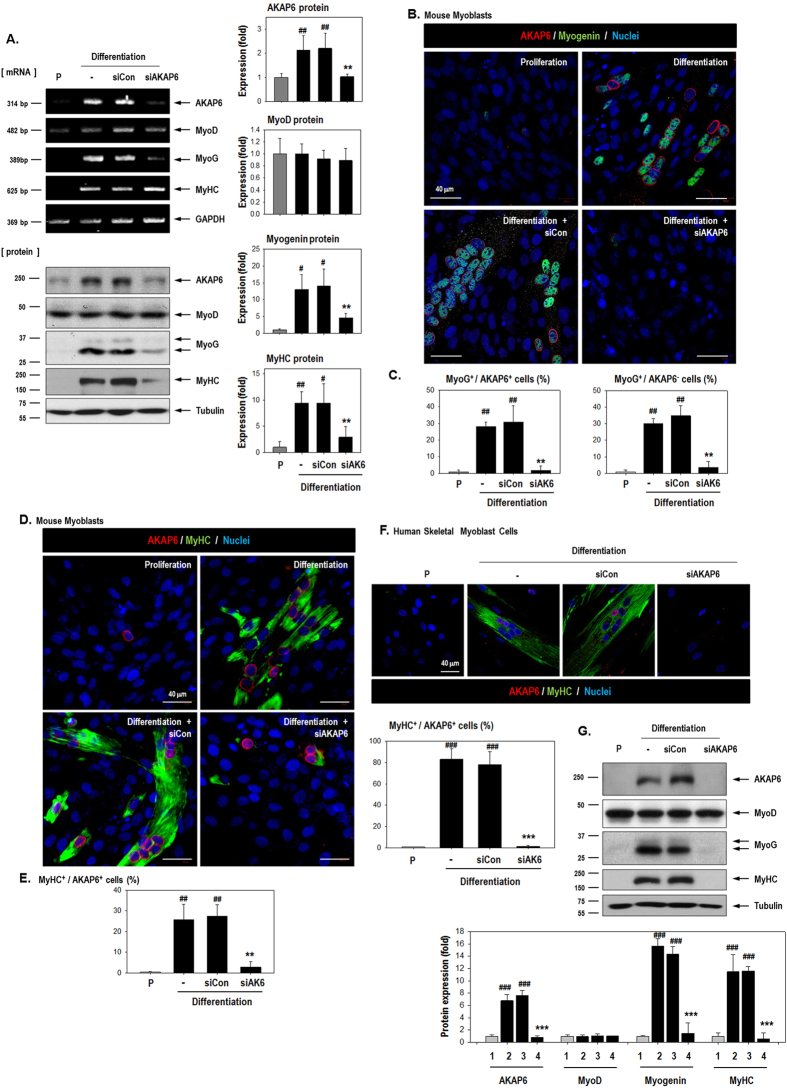
AKAP6 depletion decreases myogenin and MyHC levels and impairs myotube formation. (**A**) Inhibition of AKAP6 expression by using an AKAP6 siRNA was confirmed with RT-PCR and western blotting. C2C12 cells were transfected with a specific siRNA against AKAP6 and further incubated under differentiation conditions for 3 days. Quantification graphs for western blotting are shown. The expression was normalized to that of the proliferation group and shown as fold changes (n = 3 each, right). ^#^p < 0.05 and ^##^p < 0.01 versus proliferation; **p < 0.01 versus siCon + differentiation (one-way ANOVA). P, proliferation conditions; siCon, control siRNA; siAKAP6, siRNA against AKAP6. (**B**) C2C12 cells were transfected with siCon or siAKAP6, incubated in differentiation medium for 3 days, fixed, and immunofluorescence-stained for AKAP6 (Red) and myogenin (Green). Original magnification: ×400. (**C**) The graph shows the percentage of myogenin^+^/AKAP6^+^ cells or myogenin^+^/AKAP6^−^ cells divided by the number of nuclei (n = 5 each). ^##^p < 0.01 versus proliferation; **p < 0.01 versus siCon + differentiation (one-way ANOVA). (**D**) Immunofluorescence staining for AKAP6 (Red) and MyHC (Green) after siAKAP6 transfection in C2C12 cells. Magnification: ×400. (**E**) The graph shows the percentage for MyHC^+^/AKAP^+^ cells divided by the number of nuclei (n = 5 each). ^##^p < 0.01 versus proliferation; **p < 0.01 versus siCon + differentiation (one-way ANOVA). (**F**) Effect of AKAP6 knockdown in human myoblasts, HSMMs, under differentiation conditions. After transfection with siAKAP6, HSMMs were stained for AKAP6 (Red) and MyHC (Green). Magnification: ×400. The graph shows the percentage for MyHC^+^/AKAP^+^ cells divided by the number of nuclei (n = 5 each). ^###^p < 0.001 versus proliferation; ***p < 0.01 versus siCon + differentiation (one-way ANOVA). (**G**) Western blotting for AKAP6 and myogenic genes in HSMMs. The expression was normalized to that of the proliferation group and is shown as fold change (n = 5 each, ^###^p < 0.001 versus proliferation, ***p < 0.001 versus differentiation + siCon). 1, Proliferation conditions; 2, Differentiation conditions; 3, Diff + siCon; 4, Diff + siAKAP6.

To clarify the involvement of AKAP6 in skeletal myoblast differentiation, we inhibited AKAP6 with specific siRNA under differentiation conditions. AKAP6 siRNA inhibited AKAP6 mRNA and protein expression even under differentiation conditions (Fig. 2A). AKAP6 depletion significantly decreased myogenin and MyHC protein levels but not MyoD levels (Fig. 2A). Furthermore, AKAP6 disruption effectively blocked the formation of myotubes that stained positively for myogenin or MyHC (Fig. 2B–G). Notably, myogenin is a key transcription regulator for the terminal differentiation of muscle fibers^17^,^18^. These effects of AKAP6 depletion in mouse cells were reproduced in primary human myoblasts HSMMs (Fig. 2F,G). Together, these data suggest that AKAP6 is required for skeletal myoblast differentiation. We then used flow cytometry analysis (FACS) to test whether cell proliferation was affected by AKAP6 depletion (Fig. S4). Under differentiation conditions, the proportion of cells in the G1 phase increased, but the proportion of cells in the S phase decreased. The proportion of cells in the S phase was unaffected by siAKAP6 (Fig. S4). In addition, cell proliferation (optical density at 450 nm) as measured with a CCK-8 assay decreased under differentiation conditions but was unaffected by AKAP6 depletion (Fig. S5). These results indicate that AKAP6 depletion did not restore the proliferative potential of myoblasts under differentiation conditions, but AKAP6 depletion blocked myoblast fusion.

### shAKAP6-lentivirus impair skeletal muscle regeneration *in vivo*

**Figure 3:**
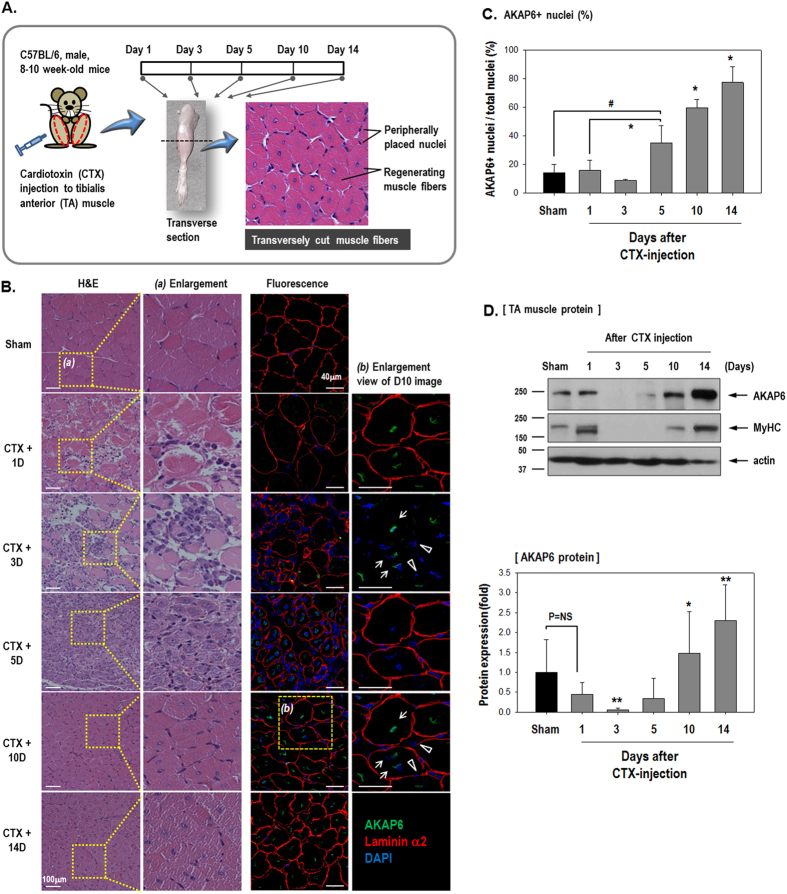
AKAP6 expression during the muscle repair period after injury. (**A**) Timeline for the *in vivo* experiment. (**B**) Hematoxylin and eosin (H&E) or immunofluorescence staining of transverse muscle sections after cardiotoxin (CTX) injection to the tibialis anterior (TA) muscle on the indicated days. Sham sections were from PBS-injected muscles. H&E staining (Magnification: ×200). Immunofluorescence staining shows that AKAP6 (Green) is predominantly observed in a centrally located nuclear envelope (arrows). Nuclei in a peripheral position in mature myofibers did not express AKAP6 (arrowheads). Laminin α2 for muscle fiber (Red), DAPI for nuclei (Blue; magnification: ×400). Immunofluorescence staining was performed in four mice using four different sections per mouse. (**C**) The graph shows the percentage of AKAP6-expressing nuclei divided by the number of nuclei (n = 5 each). ^#^p < 0.05 versus Sham; *p < 0.05 versus Day1 CTX (one-way ANOVA). (**D**) At the indicated time points, the TA muscles were frozen directly in liquid nitrogen, lysed in RIPA buffer, and analyzed with western blotting (Top). Quantification graph of the AKAP6 western blot shows fold changes from values in sham mice (Bottom, n = 4). *p < 0.05 and **p < 0.01 versus Day1 CTX (one-way ANOVA).

To test the role of AKAP6 during muscle regeneration *in vivo*, we attempted to block AKAP6 expression in muscle-injured mice during the muscle regeneration period (Fig. 3). We first observed AKAP6 expression during the muscle repair period after cardiotoxin (CTX) injury. At 10 days post-injury, the extensive presence of regenerating myofibers was identified by centrally located nuclei (Fig. 3A,B). Immunofluorescence staining with an anti-AKAP6 antibody showed that AKAP (green) was increased at days 10 and 14 (Fig. 3C). AKAP6 (green) staining was predominantly observed in the nuclear envelop of the regenerating myofibers (arrows). By contrast, AKAP6 immunofluorescence was barely detectable in peripheral nuclei in mature myofibers (arrowheads). The expression levels of AKAP6 protein in tibialis anterior (TA) muscles were similar (Fig. 3D). These data suggest that AKAP6 is highly required for regenerating myofibers.

**Figure 4:**
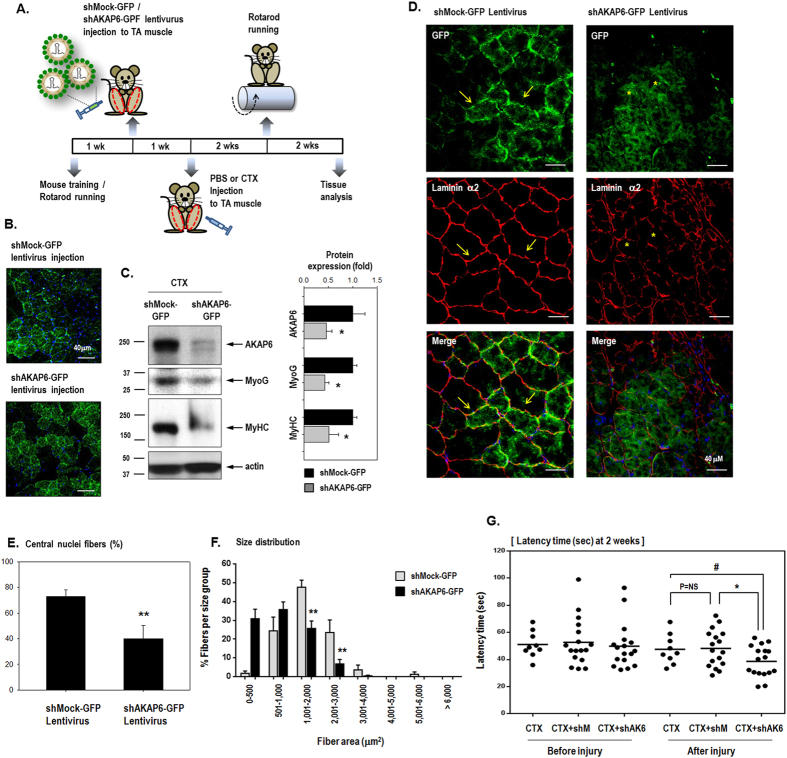
Regeneration of cardiotoxin-injected muscle is impaired by AKAP6 shRNA lentivirus. (**A**) Time table for the animal experiment a using GFP-expressing shRNA lentivirus for AKAP6 (shAKAP6-GFP) or control shRNA lentivirus (shMock-GFP) and CTX injection. (**B**) Transverse sections of muscle harvested 2 weeks after CTX injection. Muscle fibers infected by the GFP-expressing lentivirus show green fluorescence (n = 4 mice per group; magnification: ×400). (**C**) Knockdown of AKAP6 expression by the shAKAP6-GFP lentivirus was confirmed using proteins from lentivirus-infected tissues by western blotting. The quantification graph shows relative fold changes (n = 4, *p < 0.05 versus shMock-GFP). (**D**) Cross-section of TA muscle was immunostained for laminin α2 (red)/nuclei (blue) 4 weeks after CTX injection. Magnification: ×400. Muscle fibers infected with shMock-GFP or shAKAP6-GFP lentivirus fluoresced green. Skeletal muscle fibers were strongly stained by laminin α2 in the shMock-GFP lentivirus infection group (arrows). In the shAKAP6-GFP lentivirus group, muscle fibers stained with laminin α2 presented an irregular shape and were weakly detectable (asterisks; n = 4 per group). (**E**) The graph shows the percentage of central nuclei fibers divided by the number of fibers (n = 5 each, **p < 0.01). (**F**) Cross-sectional area (CSA) of the myofibers in the injured TA muscles 4 weeks after CTX injection. Myofiber CSA (μm^2^) was measured using the ImageJ software. Myofiber size in the shMock-GFP lentivirus infection group was larger than that in the shAKAP6-GFP lentivirus infection group. The shAKAP6-GFP lentivirus group showed a shift toward smaller fibers. Measurements of four different sections per mouse in four mice were used. Data are presented as mean (SEM). **p < 0.01 versus shMock-GFP (one-way ANOVA). (**G**) Rotarod test. One week before CTX injection, lentivirus was injected into the TA muscle (both legs) to induce virus amplification *in vivo*. We monitored muscle power in three groups 2 weeks after CTX injection: injury control with CTX injection (CTX only, n = 9), injury with shMock-GFP lentivirus infection (CTX+shMock, n = 17), injury with shAKAP6-GFP lentivirus infection (CTX+shAK6, n = 17). ^#^p < 0.05 versus CTX after injury; *p < 0.05 versus CTX+shM after injury (one-way ANOVA).

We determined whether muscle regeneration was affected by AKAP6 blockade (Fig. 4). GFP-expressing shRNA lentivirus for AKAP6 (shAKAP6-GFP) or non-targeting (shMock-GFP) lentivirus was injected into TA muscles followed by CTX injection (Fig. 4A). Successful lentivirus infection was observed as GFP green fluorescence (Fig. 4B), and AKAP6 knockdown by the shAKAP6-GFP lentivirus was confirmed with western blotting (Fig. 4C). Muscle fibers in skeletal muscles are surrounded by basal lamina composed primarily of laminins, which are required for myotube stability and survival^19^,^20^. Laminin α2, which is abundant in normal skeletal muscles, was expressed in healthy and regenerating myofibers. In the muscles treated with the shMock-GFP lentivirus after injury, laminin α2 expression was homogeneous throughout the muscle and strongly indicated skeletal muscle fibers with intact basal lamina (arrows, Fig. 4D). By contrast, in injured muscle treated with shAKAP6-GFP lentivirus, laminin α2 expression was weak and irregular or inhomogeneous, which indicated poorly regenerated and unhealthy muscle fibers (asterisks, Fig. 4D). We counted the centrally located nuclei, which were decreased by AKAP6 blockade (Fig. 4E). We then assessed cell death by immunofluorescence staining with a cleaved caspase-3 antibody (Fig. S6). A few myofibers were stained with the cleaved caspase-3 antibody in tissues harvested on day 5 after injury, and caspase-3 immunofluorescence was barely detectable in tissues 2 weeks post-injury. We observed no difference in caspase-3 immunofluorescence between the shMock-GFP and shAKAP6-GFP lentivirus infection groups.

The cross-sectional areas of myofibers were measured, and fiber size distribution was evaluated. Myofiber size in the shAKAP6-GFP lentivirus group showed a shift toward smaller sized fibers (Fig. 4F). These results support the conclusion that myofiber regeneration is altered in mice injected with shAKAP6-GFP lentivirus.

To monitor muscle regeneration functionally, we performed the Rotarod test after CTX muscle injury (Fig. 4G). The latency time for mice to fall off the rotating rod was measured 2 weeks after CTX muscle injury. Skeletal muscles recover naturally after CTX injury in approximately 2 weeks^21^. Consistent with histological analysis, motor function in mice infected with the shAKAP6-GFP lentivirus (CTX+shAK6) was significantly worse than that of mice infected with shMock-GFP lentivirus (CTX+shMock), supporting the importance of AKAP6 during muscle regeneration after injury.

### AKAP6 increases myogenin transcription through MEF2A, whereas myogenin up-regulates AKAP6 expression through direct binding to the AKAP6 promoter

**Figure 5:**
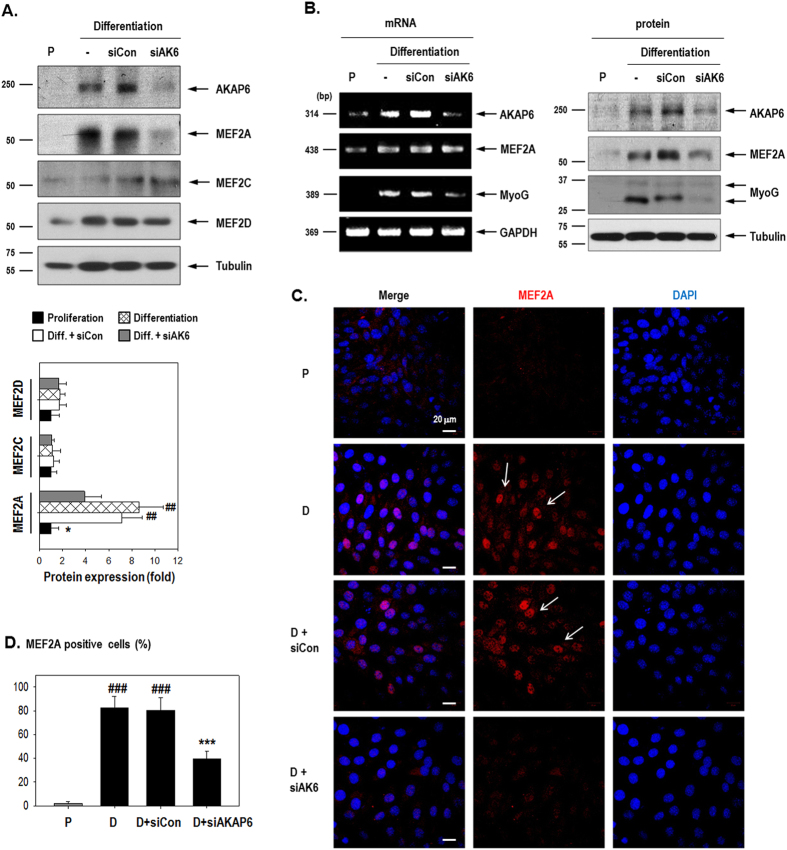
MEF2A, not MEF2C or MEF2D, mediates myogenin up-regulation by AKAP6. (**A**) C2C12 cells transfected with AKAP6 siRNA were harvested on day 3 after differentiation and western blot was performed. MEF2A was decreased by AKAP6 siRNA. Results presented in the quantification graph are expressed as a fold change from values under proliferation conditions (bottom, n = 4). ^##^p < 0.01 versus proliferation; *p < 0.05 versus siCon + differentiation (one-way ANOVA). (**B**) The induction of AKAP6 by differentiation conditions was effectively blocked by AKAP6 siRNA and both MEF2A and myogenin levels were decreased by siAKAP6. MEF2A mRNA was unaffected by AKAP6 siRNA. (**C**) Immunofluorescence staining for MEF2A (Red) after siAKAP6 transfection in C2C12 cells. Magnification: ×400. P, proliferation conditions; D, differentiation conditions; siCon, control siRNA; siAKAP6, siRNA against AKAP6. (**D**) Quantification graph of MEF2A-positive cells (percentage, n = 6). ^###^p < 0.001 versus proliferation; ***p < 0.001 versus siCon + differentiation (one-way ANOVA).

AKAP6 knockdown reduced the transcription of myogenin, a key transcription regulator for terminal differentiation of muscle fibers^17^,^18^. To determine how AKAP6 regulates myogenin transcription, we assessed MEF2 expression (Fig. 5) because MEF2 positively regulates myogenin transcription by direct binding to the myogenin promoter^22^. We determined the expression of three of the four MEF2 isoforms. MEF2A, MEF2C, and MEF2D are involved in myoblast differentiation^23^,^24^.

MEF2A and MEF2D protein levels increased under differentiation conditions, but MEF2C expression was unaffected. AKAP6 depletion decreased MEF2A protein levels but did not affect MEF2C or MEF2D expression (Fig. 5A). While MEF2A protein levels were decreased, MEF2A mRNA expression was unaffected by AKAP6 siRNA (Fig. 5B, Fig. S7), indicating that MEF2A is post-transcriptionally regulated. We then determined the cellular localization of MEF2A (Fig. 5C,D) because functional MEF2A localizes in the nucleus and acts as a transcription factor^23^. As shown in Fig. 5C, MEF2A (red) staining was remarkably increased in the nucleus (blue) under differentiation conditions. This effect was significantly decreased by AKAP6 siRNA even under differentiation conditions, suggesting that AKAP6 siRNA decreased the total amount of MEF2A protein and inhibited its nuclear localization. These data show that AKAP6 stimulates MEF2A activity, thereby influencing myogenin up-regulation for skeletal myoblast differentiation.

### Myogenin up-regulates AKAP6 expression through direct binding to the AKAP6 promoter

**Figure 6:**
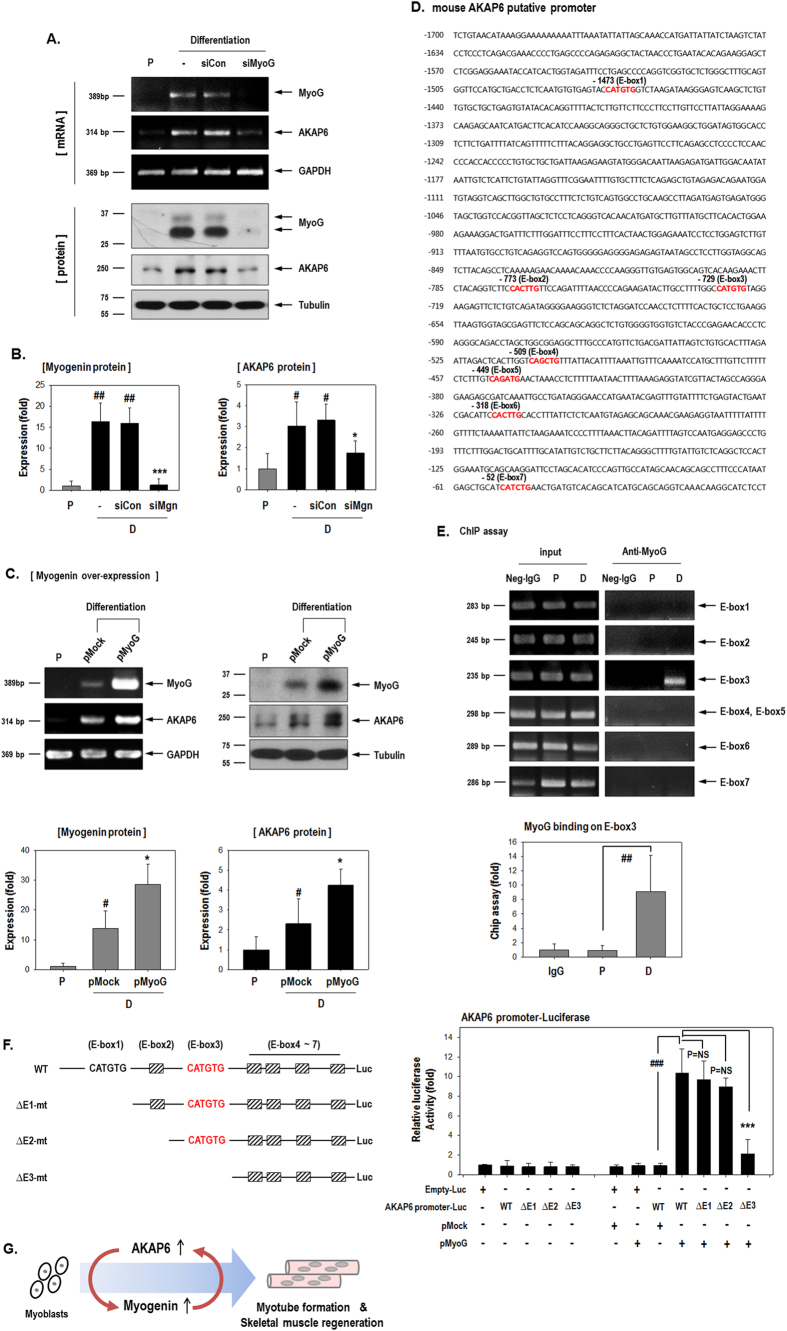
Myogenin transcriptionally increases AKAP6 by binding to an E-box on the AKAP6 promoter. (**A**) Myogenin knockdown with siMyogenin (siMyoG) abolished the differentiation effect of AKAP6 up-regulation in C2C12 cells. (**B**) Quantification graphs for western blotting show relative fold changes over values under proliferation conditions (n = 4 each). ^#^p < 0.05 and ^##^p < 0.01 versus proliferation; *p < 0.05 and ***p < 0.001 versus siCon + differentiation (one-way ANOVA). P, proliferation conditions; D, differentiation conditions. (**C**) C2C12 cells were transfected with pMyoG and incubated under differentiation conditions for 2 days. Myogenin overexpression increased AKAP6 mRNA and protein levels. Quantification graphs for western blotting show relative fold change over values under proliferation conditions (bottom, n = 5). ^#^p < 0.05 versus proliferation; *p < 0.05 versus pMock + differentiation (one-way ANOVA). (**D**) Putative promoter sequence of mouse *Akap6*. Nucleotides are numbered relative to the translation start site, and the seven E-boxes (5′-CANNTG-3′) are marked in red. (**E**) Myogenin binds to the E-box 3 site. ChIP analysis showed myogenin binding to the AKAP6 promoter. C2C12 lysates were immunoprecipitated with a myogenin antibody. The precipitated DNAs were amplified with PCR by using specific primers for the E-box (n = 5 each). Quantification graph for ChIP assay on E-box 3 region (bottom, n = 5). The graph shows the relative fold changes over levels under proliferation conditions. ^##^p < 0.01 versus proliferation (one-way ANOVA). P, proliferation conditions; D, differentiation conditions. (**F**) Luciferase assay for myogenin binding to the AKAP6 promoter. Schematic diagram of the seven E-box sites in the full-sequence AKAP6 promoter and the promoter serial deletion mutants (Left). HEK293A cells were transfected with various combinations of WT-AKAP6-promoter, AKAP6 promoter deletion mutants, and myogenin plasmids, and the luciferase assay was performed (Right, n = 3 each). ^###^p < 0.001; ***p < 0.001 (one-way ANOVA). (**G**) Schematic representation of the importance of AKAP6 in skeletal muscle regeneration.

Our results indicate that myogenin is a downstream target of AKAP6 (Fig. 2). Thus, we expected that myogenin inhibition would not affect AKAP6 expression. Unexpectedly, myogenin depletion with siRNA significantly decreased AKAP6 expression under differentiation conditions (Fig. 6A,B). Concordantly, myogenin overexpression significantly increased AKAP6 mRNA and protein expression (Fig. 6C).

In order to address the mechanism involved in the regulation of AKAP6 mRNA expression by myogenin, we first identified the AKAP6 putative promoter region by using the Ensembl program (www.ensembl.org) and cloned an approximately 1.7-kb upstream sequence from the AKAP6 transcription start site (Fig. 6D). E-box sequences (CANNTG) in the promoter region are important for the binding of basic helix-loop-helix MRFs, including MyoD, myogenin, Myf5, and MRF4, and induction of downstream muscle-specific gene expression^25^,^26^. There are seven E-box sites within the AKAP6 putative promoter and we found that the third E-box region (E-box 3) functions as a binding site for myogenin (Fig. 6E) in C2C12 cells. This selective binding to E-box 3 was notable because the sequence of E-box 1 and E-box 3 are the same (CATGTG). We confirmed myogenin binding to E-box 3 by using serial deletion mutants of the AKAP6 promoter (Fig. 6F). The luciferase activity was significantly increased after myogenin overexpression in cells transfected with the full-sequence AKAP6 promoter (WT-AKAP6-promoter). Cell transfected with the ΔE1-mt plasmid, in which E-box 1 was deleted, or the ΔE2-mt plasmid, in which E-box 2 was deleted, showed luciferase activities similar to that produced by WT-AKAP6-promoter transfection.

By contrast, the induction of AKAP6 reporter activity by myogenin was significantly inhibited in cells transfected with the ΔE3-mt plasmid in which E-box 3 was deleted. The result of this luciferase assay suggest that the induction of AKAP6 promoter activity by myogenin largely depends on the E-box 3 site and that myogenin does not bind to the E-box 1 site, which is consistent with our ChIP data. The reporter activity of the ΔE3-mt plasmid was slightly increased by myogenin, which suggested that myogenin also weakly binds to the region between E-box 4 and E-box 7, although our ChIP assay results did not show myogenin binding in these regions. Further investigations are required to determine a potential binding site in this region.

## Discussion

We demonstrated that AKAP6 is required for myoblast differentiation and skeletal muscle regeneration. AKAP6 promotes myogenin expression and myotube formation. Moreover, myogenin increases the expression of AKAP6 by binding to an E-box site on the AKAP6 promoter (Fig. 6G). The stimulation between AKAP6 and myogenin in a positive feedback loop may be a promising target for therapy after muscle injury.

AKAP6 is known to induce cardiac myocyte hypertrophy^11^ and is implicated in the proper development of cardiomyocytes^10^. Although AKAP6 is clearly involved in cardiomyocyte differentiation and cardiac function, its role in skeletal myocytes and skeletal muscle regeneration is not fully understood. Moreover, the signals that stimulate AKAP6 expression remain unclear. In this study, we found that a temporal interplay exists between AKAP6 and myogenin during skeletal myoblast differentiation. AKAP6 acts as an upstream regulator of myogenin transcription, whereas myogenin increases AKAP6 expression (Figs 2 and 6).

Myogenin, MyoD, Myf5, and MRF4 are members of the MRF family of proteins, and they show sequential expression patterns during the skeletal muscle development process^1^,^15^. Several observations suggest that these four proteins of the MRF family functionally compensate each other, at least partially, for proper myogenesis. Mice lacking both MyoD and Myf5 are born alive but die soon after birth owing to a complete absence of skeletal muscle^15^. By contrast, mice carrying mutations in either the MyoD gene or the Myf5 gene lack muscle defects^15^, indicating that MyoD and Myf5 functionally compensate each other during myogenesis. Mice with myogenin mutations die perinatally owing to a severe reduction in all skeletal muscle^27^,^28^. Thus, myogenin is considered a crucial factor for muscle development. However, MRF4 can reportedly substitute for myogenin to promote myofiber formation during the early stages of myogenesis^29^. Myogenin can also substitute for Myf5, albeit less efficiently, to determine myogenic lineage for normal rib formation^30^. Therefore, identifying the regulatory factor(s) of these MRFs might be useful for accelerating muscle regeneration.

We here found that the myogenin is upstream regulator for the induction of AKAP6 promoter activity through binding to the E-box 3 site (Fig. 6). We confirmed the AKAP6 promoter activity using C2C12 cells under proliferation and differentiation conditions (Fig. S8). The luciferase activity of WT-AKAP6-promoter under differentiation conditions was higher than that under proliferation conditions. These data suggest that the WT-AKAP6 promoter-luciferase construct active in C2C12 cells and AKAP6 promoter-luciferase activity was induced by differentiation signal. By contrast, C2C12 cells transfected with the deletion-mt plasmids including ΔE3-mt plasmid showed luciferase activities similar to that produced by WT-AKAP6-promoter. The reasons for the discrepancy in luciferase activity by the ΔE3-mt plasmid (E-box3 deletion) between HEK293A cells and C2C12 cells are unclear. However, we believe that distinctions in the regulatory factor pool may be among the contributing factors. Members of the myogenic regulatory factor (MRF) family, which can bind to E-box sequences, exist in C2C12 myoblasts under differentiation conditions and these MRFs might bind to E-box site(s) in AKAP6 promoter. Further research is needed to clarify this issue.

We established a notable molecular link between AKAP6 and myogenin in the context of muscle differentiation. We identified MEF2A as a downstream regulator of AKAP6 for myogenin expression (Fig. 5). AKAP6 (also known as muscle AKAP/mAKAP, or AKAP100) has previously been studied in C2C12 skeletal myoblasts^31^,^32^. The calcineurin/mAKAP complex regulates MEF2 signaling, which leads to skeletal and cardiac muscle differentiation^32^. AKAP6 and MEF2 bind directly, which facilitates MEF2 transcription and myogenic differentiation^31^. AKAP6/MEF2 binding increases MEF2 transcription, but a competitive inhibitor of AKAP6/MEF2 binding decreased MEF2 transcription^31^. By contrast, MEF2A mRNA expression was unaffected by AKAP6 knockdown in our repeated experiments (Fig. 5B). The reason for this discrepancy with the results of the previous report remains unclear. Vargas *et al*.^31^ observed MEF2A transcriptional activity 12 h after differentiation. We detected MEF2A mRNA on day 3 (72 h) after differentiation. We also confirmed that MEF2A mRNA expression was decreased by AKAP6 siRNA at 12 h (data not shown). The difference in MEF2A mRNA in response to AKAP6 depletion may be attributable to differences in the incubation times used to induce differentiation. Further studies are warranted to clarify this issue. Although we identified AKAP6 as an upstream regulator of myogenin expression via MEF2A, the underlying mechanism through which nuclear membrane AKAP6 controls MEF2A, which enters the nucleus, remains unknown. Further studies are required to identify the mechanism through which AKAP6 coordinates MEF2A subcellular localization during myoblast differentiation.

Scaffold proteins tether signaling enzymes and individual partner molecules to increase the efficiency of protein interaction^3^. Notably, we found that AKAP6 is a facilitator of myogenin transcription (Fig. 2) and, conversely, myogenin is an upstream stimulator of AKAP6 expression through binding to an E-box site on the AKAP6 promoter (Fig. 6). The central role of scaffold proteins is to provide a physical assembly of signaling components to increase interaction efficiency between partner molecules. AKAPs assemble upstream activators and downstream effectors within the same macromolecular complex^3^. Myogenesis and skeletal muscle regeneration are strongly coordinated by several regulatory factors. Therefore, we suggest that myogenin up-regulates the AKAP6 scaffold for efficient coordination of the signaling cascade during skeletal myoblast differentiation and myotube formation. Although we demonstrated that AKAP6 is a pivotal molecule in skeletal muscle regeneration, the implications of the emerging scaffold protein, AKAP6, being present at the nuclear membrane during muscle regeneration remains unknown. Future studies focused on identifying the components of AKAP6 complexes and their roles at the nuclear membrane in skeletal muscle are planned. These studies will provide new approaches to understanding muscle development and treating muscle diseases.

## Methods

### Myoblast culture and differentiation to myotubes

Mouse C2C12 myoblast cells (ATCC: CRL-1772) were grown in Dulbecco’s modified Eagle’s medium (DMEM; GIBCO) containing 10% fetal bovine serum (FBS; Lonza) and 1% penicillin/streptomycin (GIBCO). To induce myotube differentiation, cells over 80% confluences were cultured in differentiation media (DMEM supplemented with 2% horse serum and 1% penicillin/streptomycin). Human skeletal myoblast cells (HSMMs), human primary cells isolated from normal donors, were purchased from Lonza and cultured in SkGM-2 media (Lonza). For differentiation conditions, cells were replaced with DMEM/F12 (Lonza) with 2% horse serum. HEK293A and 293T cells (ATCC) were maintained in DMEM (GIBCO) containing 10% FBS with 1% penicillin/streptomycin. Cells were cultured at 37 °C humidified 5% CO_2_ atmosphere.

### Immunocytofluorescence staining

C2C12 mouse myoblasts or human myoblast cell HSMMs were seeded on μ-Dish^35 mm high^ (ibidi), allowed to grow up to 70–80% cell confluence, and switched to differentiation media. After differentiation, cells were fixed with 4% PFA at 4 °C for 10 min, blocked with 2% BSA-PBS solution for 1 h, and labeled with anti-AKAP6 (1:400, Covance PRB-451P), anti-myogenin (1:400, Santa Cruz sc-12732), anti-MyHC (1:400, Sigma M4276), or anti-MEF2A (1:400, Santa Cruz sc-313) overnight at 4 °C, followed by fluorescent dye conjugated secondary antibody (1:200, Invitrogen A-21202, A-31572). The nuclei were stained with DAPI (Molecular probe) and mounted using fluorescent mounting medium (DAKO). The fluorescent images were obtained using a confocal microscope (Carl Zeiss LSM710).

### Immunoblotting

C2C12 mouse myoblasts or human myoblast cell HSMMs were washed with PBS and lysed in RIPA buffer [50 mM Tris-HCl, 150 mM NaCl, 1% NP 40, 0.1% SDS, 0.5% deoxycholate, Protease Inhibitor Cocktail (Roche)]. Total protein (~20 μg) was separated in SDS-PAGE, transferred to PVDF membranes and immunoblotted with specific antibodies; AKAP6 (1:3000, Covance PRB-451P), AKAP79 (1:3000, BD 610314), MEF2D (1:3000, BD 610774), MyHC (1:3000, Sigma M4276), MEF2C (1:3000, Cell signaling #9792), AKAP-Lbc (1:3000, Santa Cruz sc-9336), AKAP12 (1:3000, Santa Cruz sc-33578), MEF2A (1:3000, Santa Cruz sc-313), myogenin (1:3000, Santa Cruz sc-12732), MyoD (1:3000, Santa Cruz sc-760), actin (1:3000, Santa Cruz sc-1616), α-tubulin (1:5000, Calbiochem CP06). After incubation with horseradish peroxidase conjugated secondary antibody (1:5000, Santa Cruz sc-2005, sc-2004, sc-2020) for 1 h, immunoreactive bands were visualized with enhanced chemiluminescence with Novex® ECL Chemiluminescent Substrate Reagent (Invitrogen).

### RNA isolation and RT-PCR

Total RNA was extracted using Trizol reagent (Invitrogen) according to the manufacturer’s instructions. Briefly, cells were collected via centrifugation and lysed in 1 mL Trizol reagent with repetitive pipetting. After incubation of the homogenized samples for 5 min at room temperature, 0.2 mL chloroform was added per 1 mL Trizol reagent. The samples were mixed vigorously and then centrifuged at 15000 rpm for 15 min at 4 °C. The RNA was precipitated from the aqueous phase by mixing with 0.5 mL isopropanol. The samples were incubated at room temperature for 10 min and centrifuged at 15000 rpm for 10 min at 4 °C. The supernatant was removed and the RNA pellet was washed once with 75% ethanol. The pellet was air-dried and dissolved in diethyl pyrocarbonate (DEPC)-treated water. RNA (1–2 μg) was converted into cDNA with a Prime 1st reverse transcriptase kit (Takara) at 42 °C for 1 h. PCR was performed with specific primers as described in Supplementary Table S1. The PCR products were visualized on 1.5% agarose gel and quantified by using GAPDH as the normalization control.

### siRNA transfection and expression plasmid transfection

For specific blockage of AKAP6 or myogenin, cells were transfected with each specific siRNA with Metafetamin pro (Biotex) 24 h prior to differentiation. Target sequence for siRNA against AKAP6 (5′-GACGAACCUUCCUUCCGAAUU-3′), MEF2A (5′– AGUAAUUAUUAGGAAUAUAA-3′) and scrambled siRNA control (non-targeting siRNA pool) are purchased from Dharmacon. The siRNA for myogenin (sc-35992) and non-targeting siRNA control (sc-37007) were obtained from Santa cruz. Myogenin full-length cDNA was amplified by PCR and cloned into pcDNA 3.1 (Invitrogen): Forward 5′- (HindIII) GGGAAGCTTATGGAGCTGTATGAGA-3′, Reverse 5′- (EcoRI) CGGGAATTCTCAGTTGGGCAT-3′. Myogenin expression vector was transfected with Metafetamin pro (Biotex).

### Cardiotoxin-muscle injury model and histological analysis

All animal experiments were performed under a protocol approved by the Institutional Animal Care and Use Committee (IACUC) of the Biochemical Research Institute at Seoul National University Hospital. This protocol complied with the NIH *Guide for ‘The Care and Use of Laboratory Animals’*. For muscle injury induction, 50 μL of 10 μM cardiotoxin (CTX, Sigma-Aldrich) or saline was administered into the right and left tibialis anterior (TA) muscles of 8- to 10-week-old male C57BL/6 mice^33^. The TA muscles were harvested at the indicated times (1, 3, 5, 10, and 14 days after CTX injection) and embedded in OCT compound or paraffin. The embedded tissues were cut in 4- to 6-μm sections, incubated with a primary antibody against AKAP6 (1:400, Covance PRB-451P), laminin α2 (1:400, Alexis Biochemical ALX-804-190-C100), and cleaved caspase-3 (1:400, Cell Signaling #9661), followed by a fluorescent dye-conjugated secondary antibody (1:200, Invitrogen A-21206, A-21247). For morphometric analysis, paraffin sections of mouse TA muscles were stained with hematoxylin and eosin (H&E) by following standard protocols. Microscopic images were obtained with an Olympus TH4-200 microscope. Myofiber cross-sectional areas (CSA; μm^2^) were measured using the ImageJ software (http://imagej.nih.gov/ij/). Randomly selected fields from four transverse-sectioned slides from four different mice were analyzed^34^,^35^.

### Lenti-virus construction for AKAP6

For specific knockdown of AKAP6 in animal study, we generated lentivirus for AKAP6. Specific AKAP6 shRNA was cloned to pLL3.7 vector (Addgene). We used double-strained hairpin oligonucleotides for AKAP6 as previously reported^36^: AKAP6 shRNA (sense strand), 5′-GACGAACCTTCCTTCCGAATTCAAGAGATTCGGAAGGAAGGTTCGTCTTTTT-3′. To package lentivirus, pLp1, pLp2, and pLp3 plasmids (Invitrogen) were transfected to HEK293T cells with pLL3.7-GFP or pLL3.7-AKAP6 using polyethylenimine (PEI, Polyscience). The transfected supernatants were harvested 2 days later, and concentrated using ultra-centrifuge (Beckman) at 4 °C, 25000 rpm for 90 min. The concentrated lentivirus pellets were re-suspended in PBS and 50 μL of lentivirus was injected into TA muscle of mice.

### Rotarod performance

We tested the exercise ability of mice after muscle injury by performing rotarod (Panlab Rota-Rods LE. 8200, Harvard Apparatus)^33^. Control shRNA or AKAP6 shRNA lentivirus were injected 7 days prior to CTX injection. After 2 weeks, mice were place on rod and we measured the latency time it takes the mouse to fall off the rod rotating under continuous acceleration (from 5 to 40 rpm). Each mouse was given three trials, measured the each latency time on the rod, and calculated the average latency time. Mice were allowed to rest for at least 5 min between each trial.

### Promoter construction and luciferase assay

We obtained putative AKAP6 promoter construct from genomic DNA of C57 mouse. 1.7-kb of DNA sequence upstream from transcriptional start site of AKAP6 gene was amplified by PCR and cloned into pGL3-basic luciferase vector (Promega). HEK293A cells were plated at a density of 3 × 10^5^ cells per well of a 6-well plate and transfected with various combinations of plasmids. After transfection, cells were lysed with Reporter Lysis Buffer (Promega), performed luciferase assay using the Luciferase Assay System kit (Promega) and a Luminometer (Promega). The normalization of transfection efficiency was determined using the β- galactosidase enzyme assay system (Promega).

### Chromatin immunoprecipitation (ChIP) assay

Chromatin immunoprecipitation (ChIP) assay for myogenin was performed with ChIP assay kit (Upstate)^37^. Mouse AKAP6 promoter vector was transfected into C2C12 myoblasts and lysates were immunoprecipitated with anti-myogenin antibody (Santa cruz sc-12732X) and PCR was performed with the primer designed to amplify a region of the AKAP6 promoter harboring 7 putative myogenin binding region (Supplementary Table S2).

### Statistical analysis

Quantification of band intensity was performed by Image J software (NIH, Bethesda, MD, USA) and normalized to the intensity of internal control. All results are expressed as means ± standard deviations (SD). The differences between the groups were compared by one-way analysis of variance (ANOVA) followed by post hoc analysis. P values < 0.05 were considered statistically significant.

## Additional Information

**How to cite this article**: Lee, S.-W. *et al*. AKAP6 inhibition impairs myoblast differentiation and muscle regeneration: Positive loop between AKAP6 and myogenin. *Sci. Rep.* **5**, 16523; doi: 10.1038/srep16523 (2015).

## Supplementary Material

Supplementary Information
