# Supplementary material for: AKAP6 inhibition impairs myoblast differentiation and muscle regeneration: Positive loop between AKAP6 and myogenin
Source: Sci Rep. 2015 Nov 13;5:16523. doi: 10.1038/srep16523 (PMC4643297; doi:10.1038/srep16523)

AKAP6 inhibition impairs myoblast differentiation and muscle regeneration: Positive loop between AKAP6 and myogenin

Sae-Won Lee[1,*], Joo-Yun Won[1,*], Jimin Yang[1], Jaewon Lee[1], Su-Yeon Kim[1], Eun Ju Lee[1], Hyo-Soo Kim[2,3]

[1]Biomedical Research Institute and IRICT, Seoul National University Hospital; [2]Department of Internal Medicine and IRICT, Seoul National University Hospital, Seoul; [3]Department of Molecular Medicine and Biopharmaceutical Sciences, Graduate School of Convergence Science and Technology, Seoul National University, Korea

(*S-W.L. and J-Y.W contributed equally to this work.)

Corresponding author:

Hyo-Soo Kim, MD, PhD

Professor, Department of Internal Medicine, Seoul National University Hospital,

Director, National Research Laboratory for Cardiovascular Stem Cell,

101 Daehak-ro, Jongno-gu, Seoul 110-744, Korea.

Phone: 82-2-2072-2226; Fax: 82-2-766-8904;

E-mail: hyosoo@snu.ac.kr or usahyosoo@gmail.com

Supplementary Materials

Supplementary Methods

Figure S1- S8

Table S1, S2

Supplementary Methods

Cell cycle assay by flow cytometry

To checked the effect of AKAP6 depletion on the cell cycle, C2C12 cells (3 x 10^5) were transfected with siRNA against AKAP6 and differentiated for 3 days. Cells harvested with trypsin-EDTA, washed with PBS containing 5mM EDTA, and fixed in 70% ethanol at -20 °C. After 24 h, the cells were collected by centrifugation and resuspended in PBS containing 5mM EDTA. After 50μg/mL RNase A digestion, the cells were stained with 50 μg/mL propidium iodide (PI, sigma) for at least 30 min before analysis by flow cytometry instrument (Becton Dickinson). Data were analyzed with the ModFit software (Verity Software House).

Cell proliferation assay

C2C12 cells (3 x 10^5) were seeded on 6-well plate, transfected with siRNA and induced differentiation. At 3days on differentiation, cell proliferation was analyzed using Cell Counting Kit-8 (CCK-8, Dojindo) according to manufacturer's instructions. CCK-8 was treated into C2C12 medium. After incubation for 3 h in the dark, 100 μL□media were

obtained and the absorbance was measured at 450 nm using an ELISA reader.

Supplementary Figure legends

Supplementary Fig. S1. Only AKAP6 is up-regulated during skeletal myoblast differentiation (a) Morphological change of C2C12 myoblast cells was observed by phase-contrast microscope. Myotube formation (arrows) was increased upon differentiation (Magnification: ×100). **(b, c)** The expression pattern of AKAP family proteins related to muscle was observed by western blotting. Quantification graph show relative fold change compared with levels on day 1 under proliferative conditions (n = 3 each, #p < 0.05 versus proliferation on day 1, *p < 0.05 versus differentiation on day 1).

Supplementary Fig. S2. Quantification graphs of Fig. 1A. Graphs show relative fold change compared with levels on day 1 under proliferative conditions (n = 4 each, western blotting; n = 3 each, RT-PCR; ###p < 0.001 versus proliferation on day 1, ***p < 0.001 versus differentiation on day 1).

Supplementary Fig. S3. C2C12 cells were stained with AKAP6 (Red) and myogenin (Green). AKAP6 showed clear immunofluorescence in nuclear envelope and myogenin is in nucleus (Magnification: ×400). MyoG+/ AKAP6- (arrows) or MyoG-/ AKAP6+ (arrowheads) were shown.

Supplementary Fig. S4. The cell cycle was analyzed by FACS (a) According to the amount of DNA, cell cycle points were assigned to G0/G1 phase, S phase and G2/M phase. **(b)** Quantitative graph of FACS analysis from four-different experiments was shown ($n = 4$ each, $*p < 0.05$ versus proliferation group).

Supplementary Fig. S5. Proliferation assay after depletion of AKAP6 in C2C12 cells. C2C12 cells were transfected with siCon or siAKAP6, incubated in differentiation media for 3 days. Cell proliferation was analyzed using Cell Counting Kit-8, and optical density at 450 nm was measured ($n = 4$, $*p < 0.05$ versus proliferation group).

Supplementary Fig. S6. Immunofluorescence staining for cleaved caspase-3. Cross-section of TA muscle was immunostained for cleaved caspase-3 (Red)/ laminin α2 (White)/ nuclei (Blue). Tissues of 5 days and 2 weeks after CTX injection were used. Magnification: x400 ($n = 4$ each group). Cleaved caspase-3 was stained at day 5 tissue (arrows), and caspase-3 immunofluorescence was barely detectable in tissues 2 weeks post-injury. Caspase-3 immunofluorescence (arrows) showed no difference between shMock-GFP and shAKAP6-GFP lentivirus infection group.

Supplementary Fig. S7. Quantification graphs of Fig. 5B. Graphs show relative fold change compared with levels on day 1 under proliferative conditions ($n = 4$ each, $\#\#\#p < 0.001$ versus proliferation, $***p < 0.001$ versus differentiation + siCon).

Supplementary Fig. S8. Luciferase assay with the AKAP6 promoter in C2C12 cells under

proliferation and differentiation conditions. Cells were transfected with various combinations of WT-AKAP6-promoter and AKAP6 promoter deletion mutants, and the luciferase assay was performed (n = 3 each). ###p < 0.001 versus proliferation + Empty-Luc; ***p < 0.001 versus differentiation + Empty-Luc; §§p < 0.01 versus differentiation + AKAP6 promoter-Luc (one-way ANOVA).

Supplementary Table S1. Primer sequence for RT-PCR

Primer		Sequence	Size(bp)	Tm, Cycle
AKAP6	Forward	TCTGGGGACATAAGTGTGAG	314	Tm: 55℃
	Reverse	CCTGAATGATGCGTTGGACT		Cycle: 27
Myogenin	Forward	GCGCAGGCTCAAGAAAGTGAAT	389	Tm: 60℃
	Reverse	GTTGAAGTCGCAGGAGACAAC		Cycle: 30
MyoD	Forward	CATCCGCTACATCGAAGGTC	482	Tm: 55℃
	Reverse	TCGCATTGGGGTTTGAGCC		Cycle: 27
MyHC	Forward	AGAAGGAGGAGGCAACTTCTG	625	Tm: 55℃
	Reverse	ACATACTCATTGCCGACCTTG		Cycle: 27
MEF2A	Forward	ATGGGGCGAAAGAAGATACAA	432	Tm: 57℃
	Reverse	GACTGTGACAGACATTGAGAAGTT		Cycle:27
MEF2C	Forward	GCTTTGAGATGCCAGTTACCA	308	Tm: 50℃
	Reverse	GGGAGGAGATTTGGCTTGTA		Cycle:30
MEF2D	Forward	CCCTGAGGAAGAAGGGTTTC	251	Tm: 60℃

	Reverse	ATGTCACCAGGGAAGGAGTG		Cycle:27
GAPDH	Forward	CATGACAACTTTGGCATTGTG	369	Tm: 65℃
	Reverse	GTTGAAGTCGCAGGAGACAAC		Cycle:25

Supplementary Table S2. Primer sequence for ChIP assay

ChIP primer		Sequence	Size(bp)	Tm, Cycle
E-box 1	Forward	GCTACTAACCCTGAATACACAG	283	Tm: 60℃
	Reverse	CTATCCAGCCTTCCACAGAG		Cycle:30
E-box 2	Forward	CACACTGGAAAGAAAGGACTG	245	Tm: 60℃
	Reverse	GTTCTGGGGTTAAAATCTGG		Cycle:30
E-box 3	Forward	CCAGATTTTAACCCCAGAAG	235	Tm: 60℃
	Reverse	AGTGCACAGACTAATAATCG		Cycle:30
E-box 4-5	Forward	TCCTGAAGGTTAAGTGGTAG	298	Tm: 55℃
	Reverse	CCCTATCAGGCAATTTGATC		Cycle:30
E-box 6	Forward	ACTAGCCAGGGAGAAGAGCGATCA	289	Tm: 60℃
	Reverse	GCTGCATTTCCAGTGGAGCCT		Cycle:30
E-box 7	Forward	GTAGAGCAGCAAACGAAGAGG	286	Tm: 55℃
	Reverse	CCTTGTTTGACCTGCTGCATG		Cycle:30

Supple Figure S1.

a.

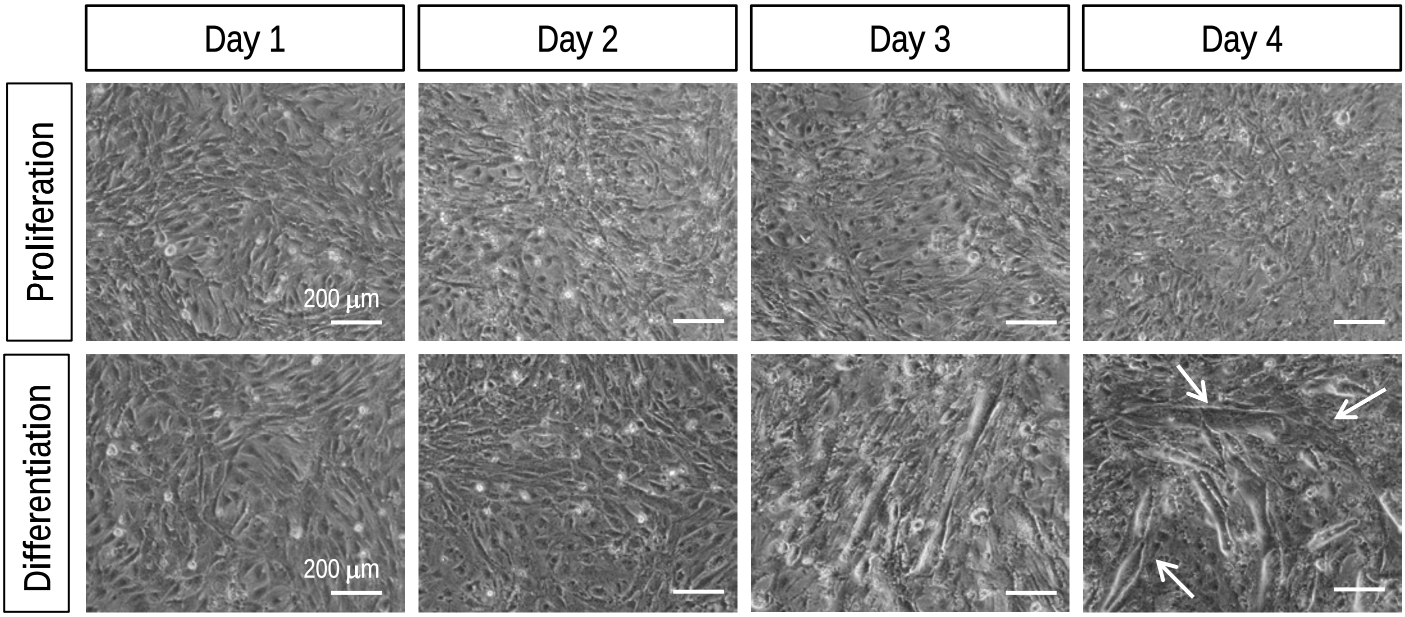

b.

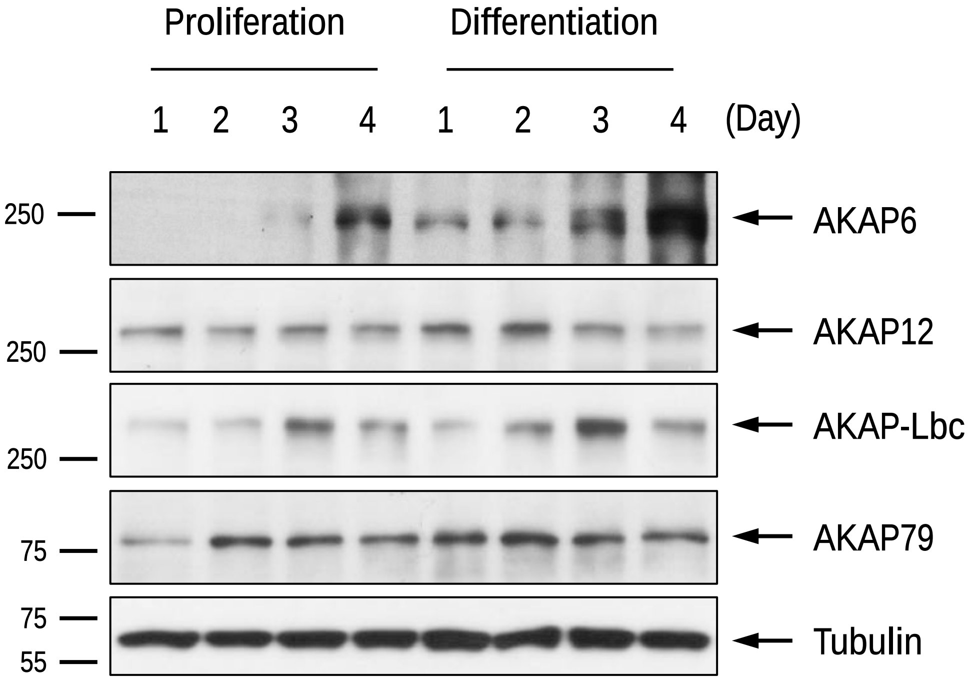

Supple Figure S1-cont'd.

c. Quantification graph of western blot

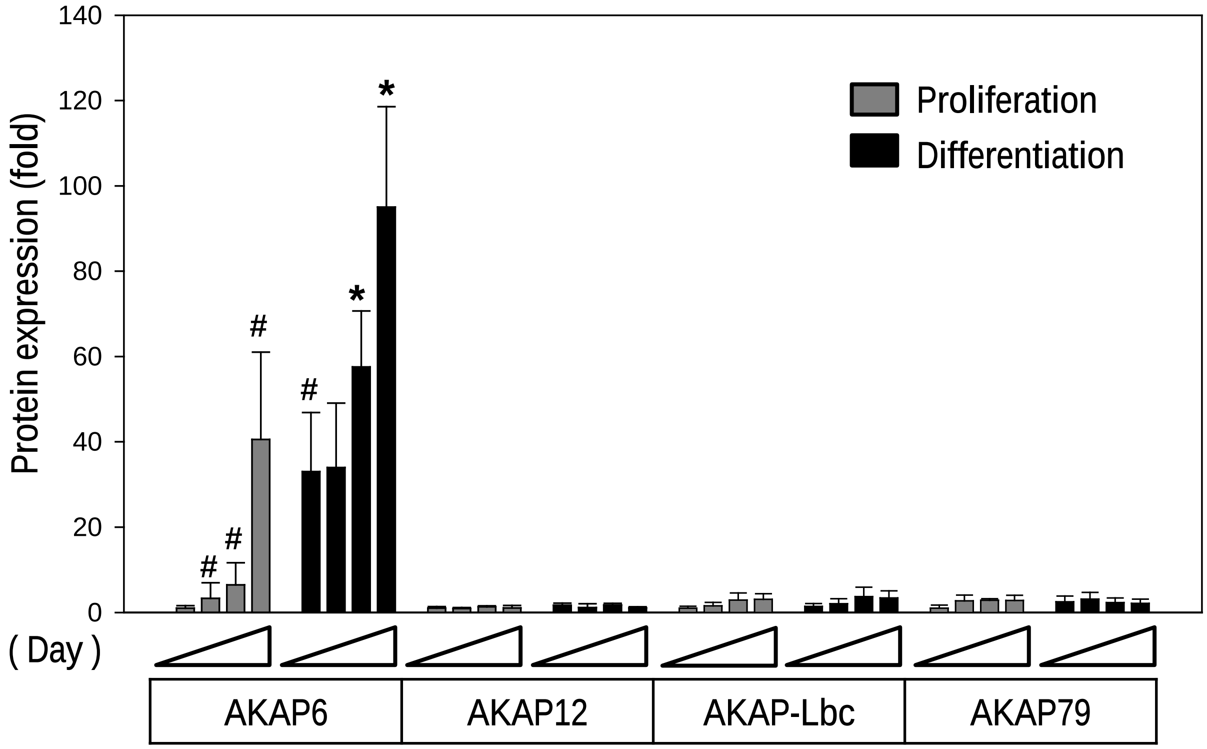

Supple Figure S2

a. Quantification graphs for western

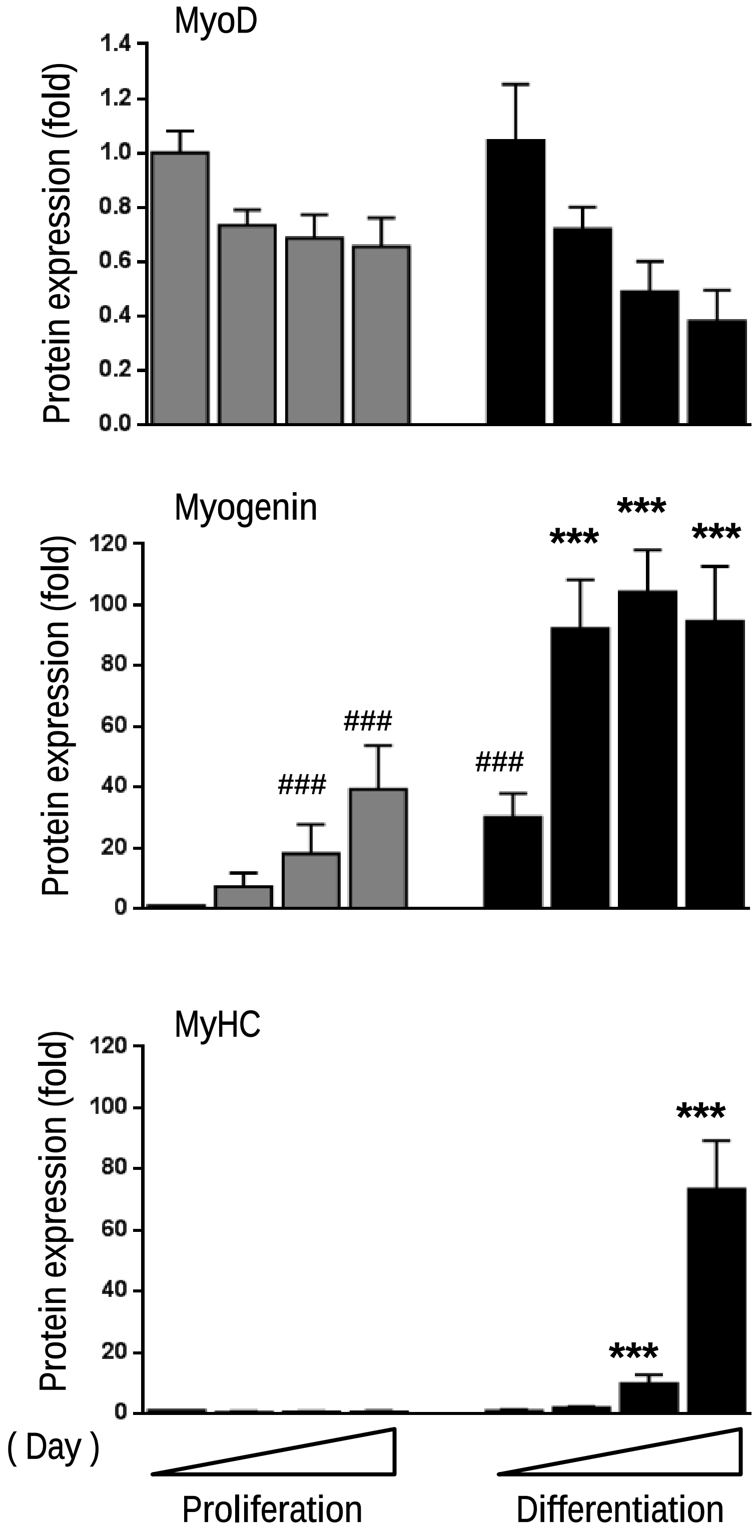

Supple Figure S2-cont'd.

b. Quantification graphs for RT-PCR

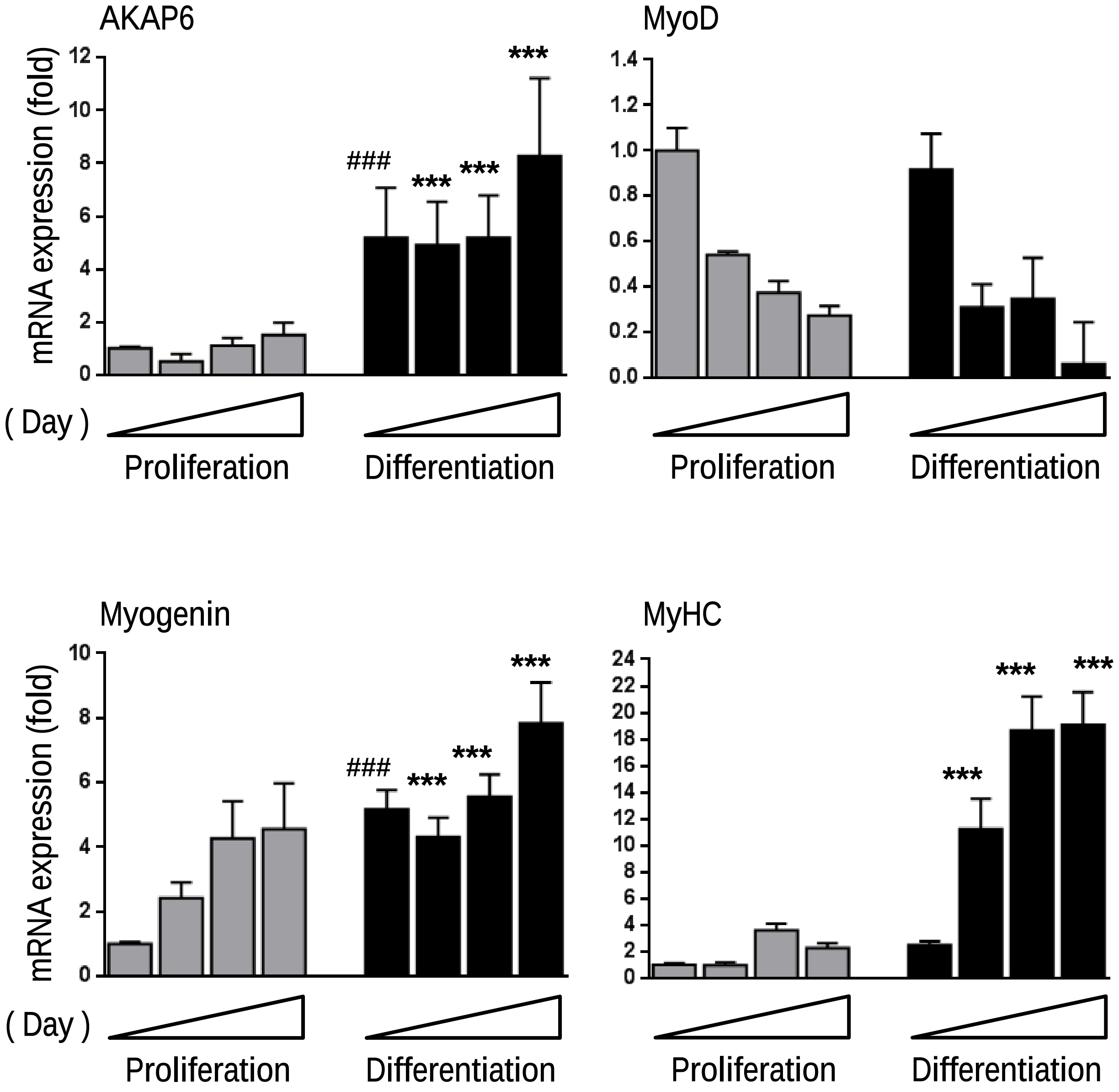

Supple Figure S3

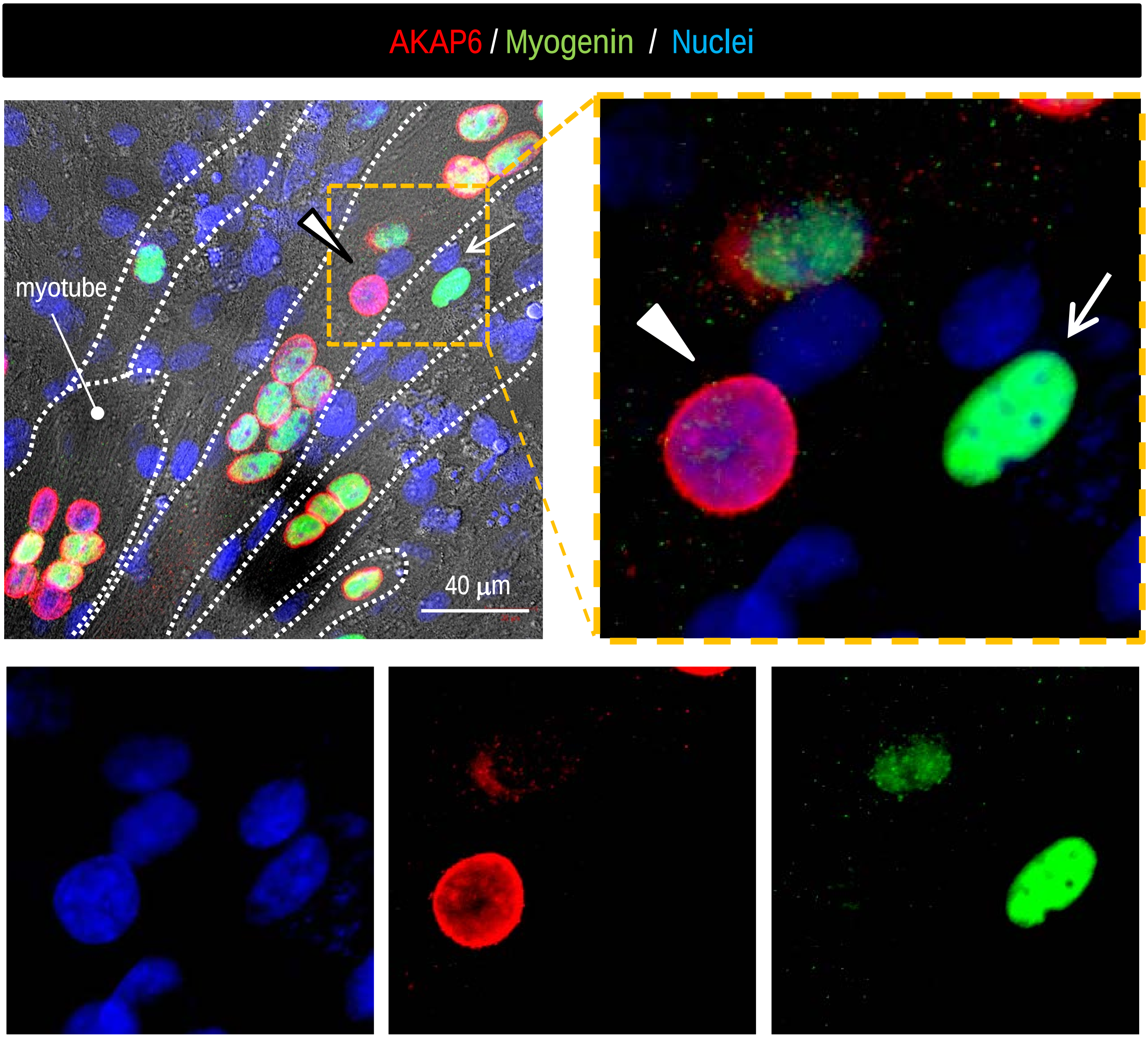

Supple Figure S4

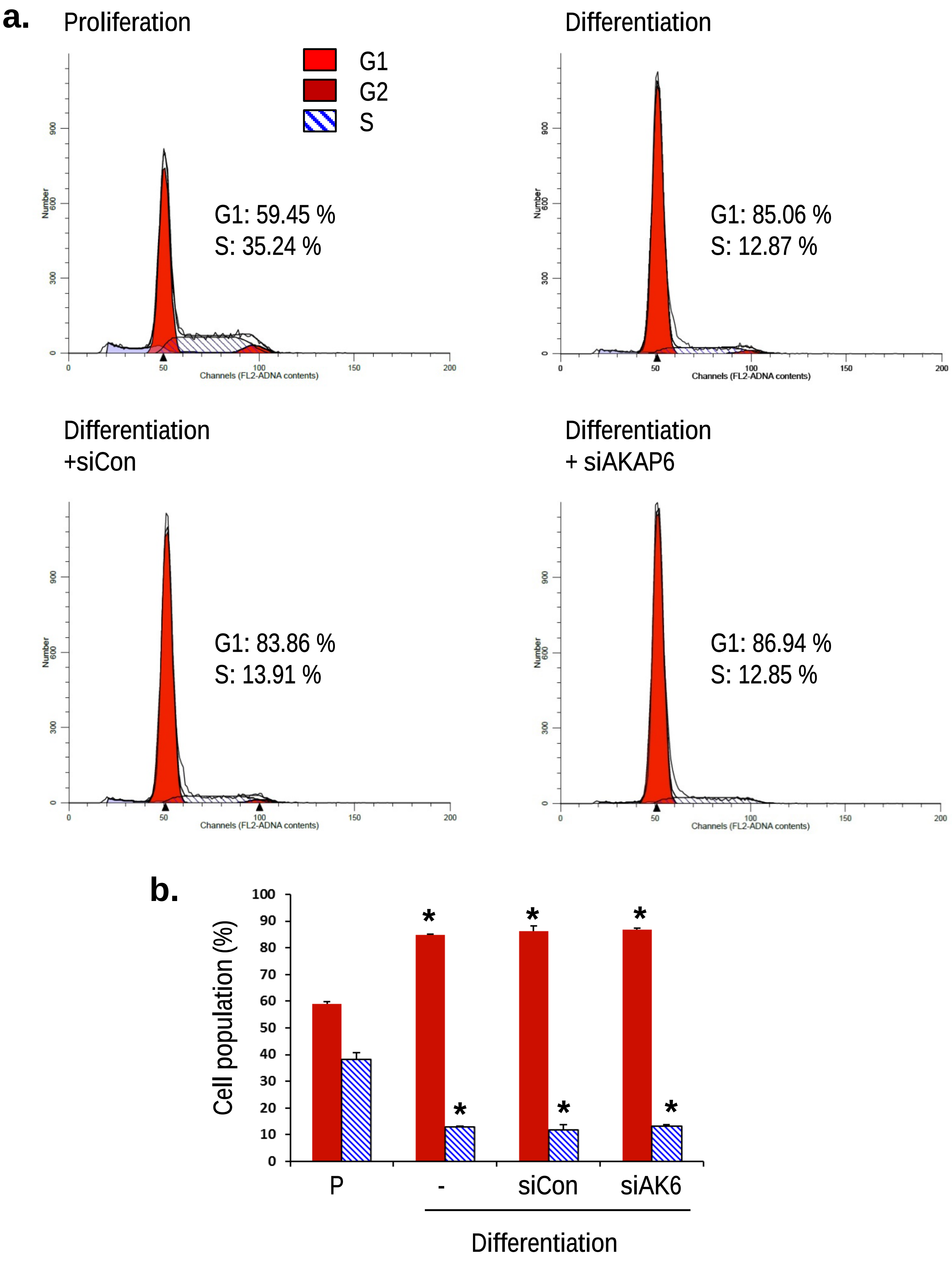

Supple Figure S5

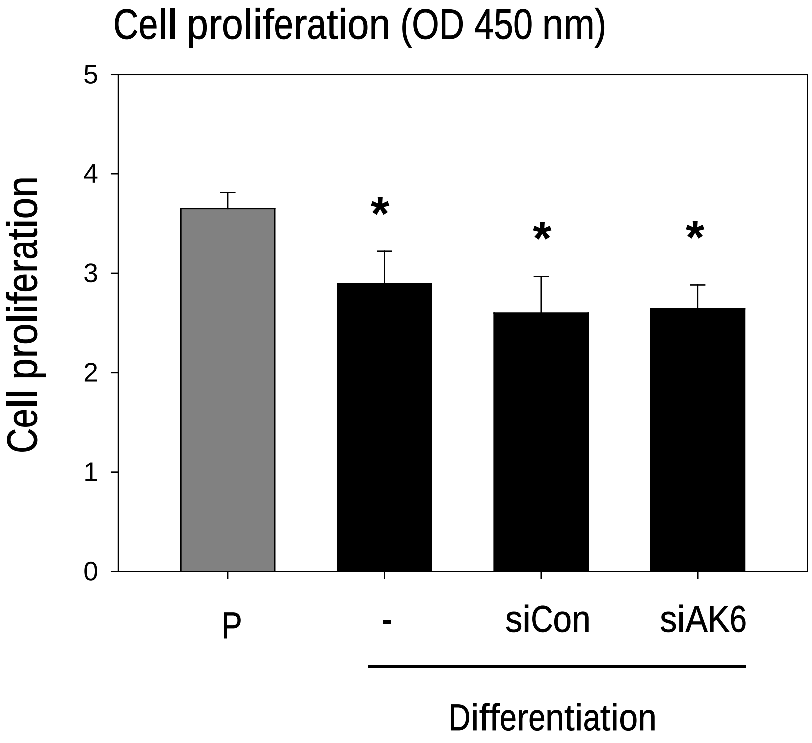

Supple Figure S6

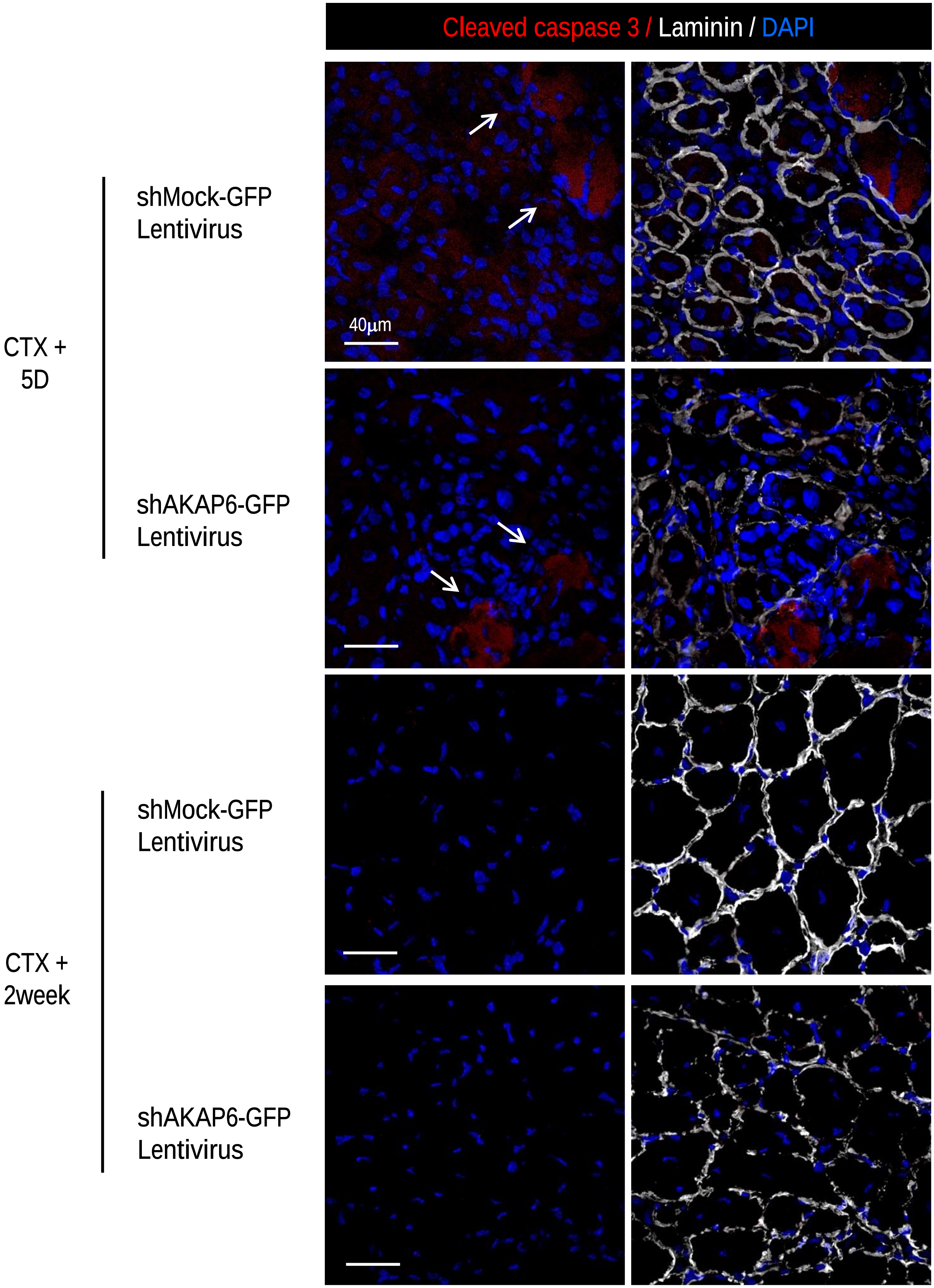

Supple Figure S7

a. Quantification graphs for RT-PCR

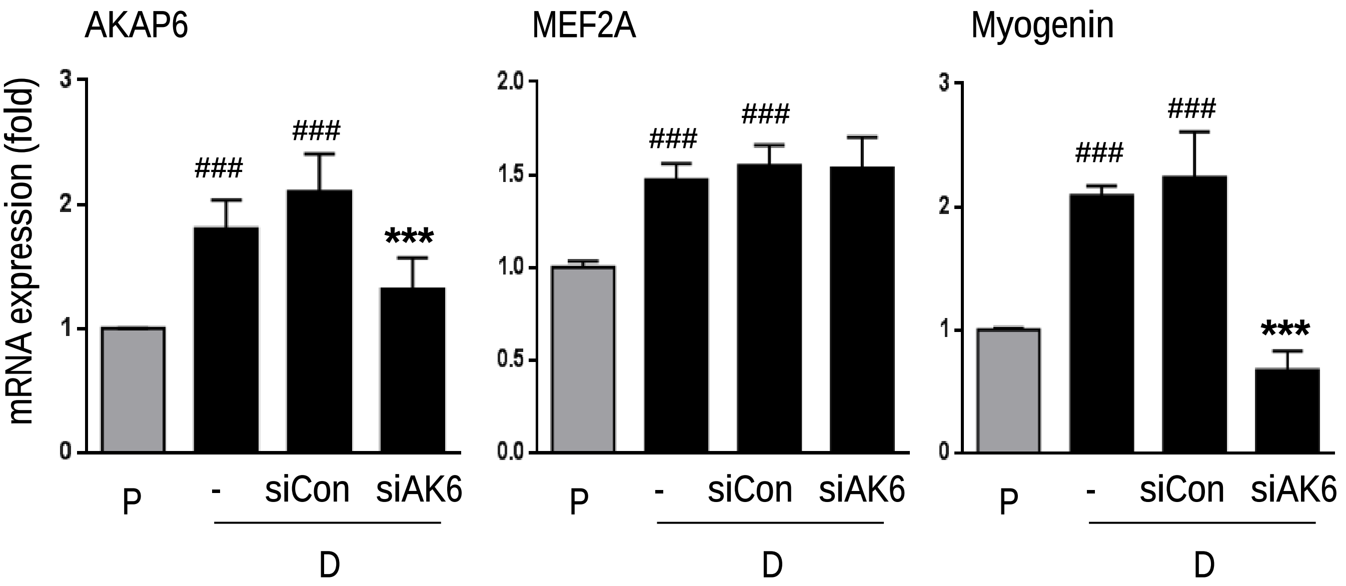

b. Quantification graphs for western

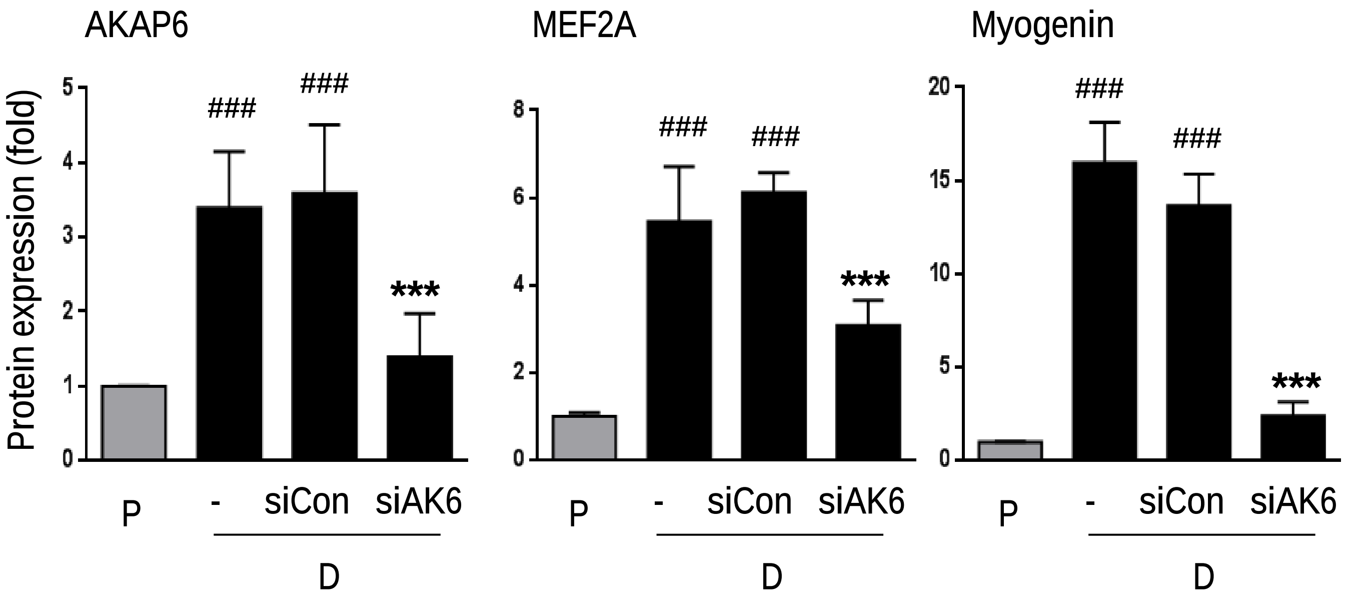

Supple Figure S8

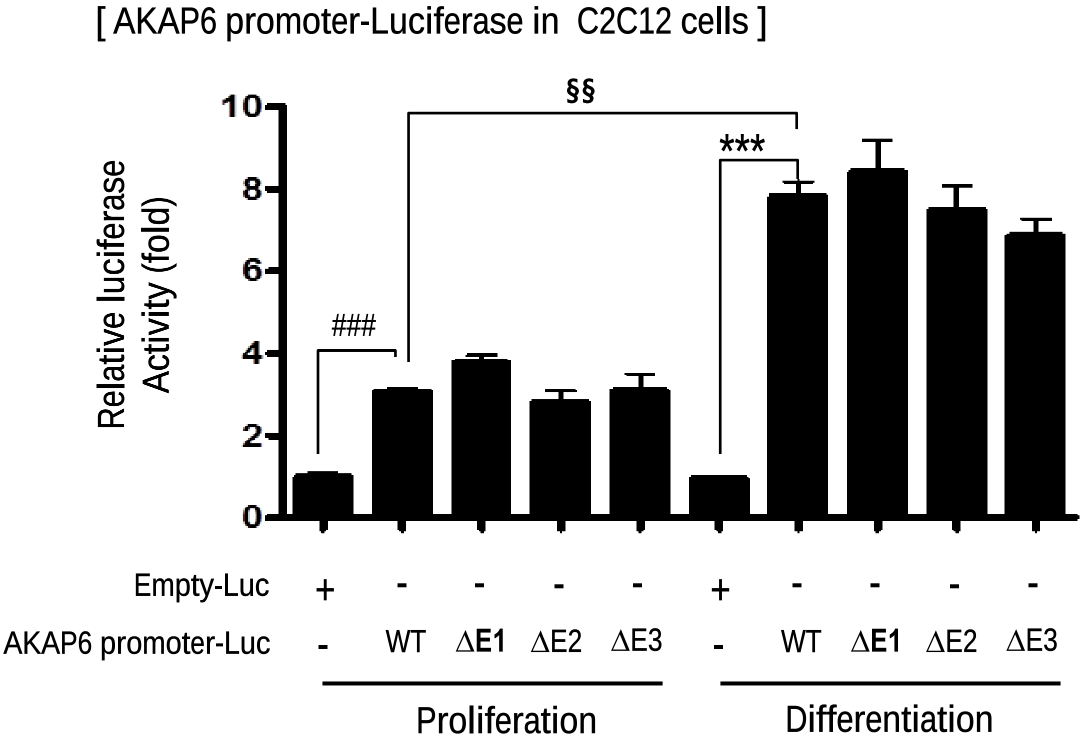

Supplement: Supplementary Information [file srep16523-s1.pdf]
